# Mandarin-English Bilinguals Process Lexical Tones in Newly Learned Words in Accordance with the Language Context

**DOI:** 10.1371/journal.pone.0169001

**Published:** 2017-01-11

**Authors:** Carolyn Quam, Sarah C. Creel

**Affiliations:** 1 Center for Research in Language, University of California, San Diego, La Jolla, California, United States of America; 2 Departments of Speech, Language, and Hearing Sciences and Psychology, University of Arizona, Tucson, Arizona, United States of America; 3 Department of Speech & Hearing Sciences, Portland State University, Portland, Oregon, United States of America; 4 Department of Cognitive Science, University of California, San Diego, La Jolla, California, United States of America; Max Planck Institute for Human Cognitive and Brain Sciences, GERMANY

## Abstract

Previous research has mainly considered the impact of tone-language experience on ability to discriminate linguistic pitch, but proficient bilingual listening requires *differential* processing of sound variation in each language context. Here, we ask whether Mandarin-English bilinguals, for whom pitch indicates word distinctions in one language but not the other, can process pitch differently in a Mandarin context vs. an English context. Across three eye-tracked word-learning experiments, results indicated that tone-intonation bilinguals process tone in accordance with the language context. In Experiment 1, 51 Mandarin-English bilinguals and 26 English speakers without tone experience were taught Mandarin-compatible novel words with tones. Mandarin-English bilinguals out-performed English speakers, and, for bilinguals, overall accuracy was correlated with Mandarin dominance. Experiment 2 taught 24 Mandarin-English bilinguals and 25 English speakers novel words with Mandarin-like tones, but English-like phonemes and phonotactics. The Mandarin-dominance advantages observed in Experiment 1 disappeared when words were English-like. Experiment 3 contrasted Mandarin-like vs. English-like words in a within-subjects design, providing even stronger evidence that bilinguals can process tone language-specifically. Bilinguals (N = 58), regardless of language dominance, attended more to tone than English speakers without Mandarin experience (N = 28), but only when words were Mandarin-like—not when they were English-like. Mandarin-English bilinguals thus tailor tone processing to the within-word language context.

## Introduction

Different languages use different acoustic-phonetic dimensions to contrast words. This creates a challenge for bilingual listeners. It would be sub-optimal for a bilingual listener to weight acoustic dimensions the same way when processing sounds and words in both languages, because they would either pick up on spurious differences or ignore important ones. Thus, in theory, the ideal bilingual listener would weight acoustic dimensions in accordance with the phonological structure of each language. Yet in practice, the extent to which such context-specific processing takes place is unclear.

One particularly underexplored spoken-language feature that varies across languages is linguistic pitch or *lexical tone*. The linguistic functions of pitch variation vary substantially across languages. In English, an *intonation language*, a rising pattern can indicate a yes/no question, while a fall can convey a statement. In Mandarin, a *tone language*, pitch contours differentiate words. Mandarin has four primary lexical tones—high flat (tone 1), rising (tone 2), low dipping (tone 3), and falling (tone 4)—realized over the domain of the syllable. Mandarin syllable structure is fairly limited [1], allowing for roughly 400 segmental combinations [2], but adding lexical tones quadruples the number of potential syllables. The syllable ‘ma,’ for example, can mean *horse*, *mother*, *hemp*, or *to scold* depending on its tone [3]. For a Mandarin speaker, then, exploiting lexical pitch facilitates word identification. In English, by contrast, listeners must recognize words *despite* changes in pitch contour, so treating pitch as lexical would be detrimental. Thus, it would be advantageous for Mandarin-English bilinguals to process word-level pitch in accordance with the language context.

The present study asks whether bilinguals who speak both a tone language (Mandarin) and an intonation language (English) can match their processing of tones in newly learned words to the phonology of the language they are currently hearing. In the sections below, we review two questions that are relevant to this topic: (1) Does existing evidence suggest that bilinguals can match processing of segmental phonological contrasts to the language context? and (2) Given what we know about how experience shapes pitch processing, might we predict that bilinguals can match their processing of linguistic pitch to the language context?

### Do bilinguals tailor phonetic processing to the language context?

Our investigation of whether bilinguals can process pitch in accordance with the language context speaks to a larger theoretical debate. Once their language systems are established, bilinguals might experience interference between representations of their two languages, such that phonetic-processing strategies from their native or dominant language inevitably “bleed over” into the other language ([4–6]). Alternatively, bilinguals might process acoustic information in accordance with the particular language they are hearing [7–10], by using context to up- or down-regulate their use of phonetic dimensions.

While no research has considered the question of context-specific encoding of lexical tone information, previous studies have found language-specific processing of *consonants* in bilinguals. Bilinguals use a different perceptual boundary for voice-onset time when processing words in English vs. languages with different boundaries (e.g., Spanish: [8]; Dutch: [9]; and French: [10]). However, these earlier studies were also consistent with *temporary* perceptual boundary shifts that can be seen even in monolinguals (see, e.g., [11–13]), rather than actual storage of two separate boundaries. Recently, Gonzales and Lotto [7] demonstrated that bilinguals—but not monolinguals—imposed different VOT boundaries for initial consonants (b or p) based on whether an English or Spanish “r” occurred in the second syllable of a nonsense word. This finding suggests that bilinguals do indeed store and access two different phoneme boundaries for stop consonants, and can use single-word phonetic context to infer the relevant language.

This investigation necessarily entails the question, *what counts as a language context*? The literature on context-dependence of memory (e.g., [14]) indicates that memories can be linked to the physical surroundings in which they were encoded. For language, context might include one’s interlocutor and their preferred language, the language of the current conversation (see [15] for language-dependent autobiographical memory), or surrounding words in a sentence. Another construal of context is sound-based: acoustic-phonetic or phonotactic cues within a sentence, or even in a particular word itself [7], might evoke one language over another. Word-specific phonetic information could be an important cue to a word’s language membership, especially in language situations with frequent code switching. This account predicts that the *within-word* phonetic/phonological content might be more important for phonological processing than the extra-word context.

### Should we expect language-specific tone processing?

The present work is one of the first (with [16]) to extend investigations of context-based phonetic processing in bilinguals to lexical tones. What might we predict for lexical tones, given what we know about how experience shapes pitch processing? At first glance, the literature on experience-dependent pitch processing might seem to suggest that bilinguals should *not* be able to shift their processing. Tone-language experience shapes tone discrimination by early infancy [17, 18], leading to more accurate pitch tracking at the auditory-brainstem level [19], different preattentive cortical processing of tones [20], and more categorical processing of tones [21] than seen in English speakers. Related evidence suggests that adult listeners’ pitch-processing abilities in language generalize to music ([22, 23]; but see [24]) and that pitch processing abilities in music generalize to language ([25–29]). If pitch processing is shared across domains (music to language and vice versa), then it is plausible that it is shared across a listener’s languages. Thus, it might appear that listeners are either adapted for tone processing or for intonation-language processing (see also [30]), and that these perceptual differences are preattentional and highly automatized.

It is plausible, however, that processing of pitch contours during *word recognition* could still be adapted to the language context. Discrimination vs. higher-level processing of pitch structure for a particular language group may not pattern together, and pitch sensitivity seems to be impacted by the particular linguistic function the pitch variation is serving ([31, 32]; see also [33]). Additionally, bilinguals can use different phonetic boundaries for consonants to process languages like English and Spanish. Thus, efficient, language-specific processing of tone does not necessarily imply that bilinguals are “stuck” in a single mode of pitch processing.

In addition, there are limits to the degree to which pitch-processing strategies generalize across domains. First, neural facilitation for pitch processing, e.g., for Chinese speakers processing musical pitch, does not necessarily translate into perceptual advantages [34]. Second, even when perceptual advantages are found, this does not necessarily imply that these listeners would always attend more to pitch than their counterparts with less pitch experience. For example, English-speaking musicians must surely attend to pitch differently when listening to music vs. English speech. Likewise, generalization of pitch experience between music and language does not necessarily imply that generalization is obligatory for bilinguals who have experience with two languages that use pitch differently. Thus, despite some evidence that pitch-processing strategies generalize across domains like music vs. language, proficient bilinguals might still optimally match their pitch processing to tone vs. non-tone language input. Supporting this notion, recent work [16] found that Mandarin-English bilingual 4- to 5-year-olds were more sensitive to changes in newly learned words’ tones in a Mandarin context than in an English context.

### The current study

We investigated the influence of language context on tone processing in Mandarin-English bilinguals. We taught novel tone-bearing words (i.e., pseudowords) referring to novel pictures, in either a Mandarin or English context. After teaching word-object pairings, we employed a visual-world paradigm in which we tracked participants’ gaze to two pictures as they identified and clicked on the referent of the target word in real time.

Across three experiments, we investigated different instantiations of language context. In Experiment 1, listeners learned novel words for novel objects that contained Mandarin tones and were phonologically potentially compatible with Mandarin. We were interested in whether the global language context—the language bilinguals are hearing across and within sentences and throughout the experiment—might cue bilinguals that they are in an English vs. a Mandarin language context, and cause them to encode tone information in novel words differently.

To test the role of word-specific context, in Experiment 2, listeners learned words that contained Mandarin tones, but that were phonetically and phonologically English-like, in contrast to the Mandarin-compatible words from Experiment 1. Experiment 3 further investigated within-word context with a carefully-matched set of words in a within-subjects design, by teaching each participant both a set of Mandarin-like words and a set of English-like words, all of which contained Mandarin tones. For both types of language context, we tested whether Mandarin-English bilinguals would exploit tone more to disambiguate newly learned words when they were presented in a Mandarin context vs. an English context. Bilinguals’ ability to use tone in a Mandarin-like manner was gauged by comparison to monolingual English-speaking participants, who are capable of learning something about tones ([27, 35]), but cannot do so to the extent of an actual tone-language speaker. Across experiments, phonological processing of newly learned words was assessed via both clicking accuracy (a discrete choice) and gaze (a time-course measure). The accuracy measure indicates what proportion of the time participants *failed* to identify the correct referent. The gaze measure indicates listeners’ degree of difficulty in identifying the word even when they are eventually successful, and is less susceptible to ceiling effects than accuracy.

## Language-Dominance Assessments

Across all three experiments reported in this paper, Mandarin-English bilinguals’ relative language dominance was assessed via three measures. (1) The Multilingual Naming Test (MINT; [36]) is an evaluation of bilingual vocabulary. The MINT score in each language is the number of pictures named correctly (out of 68). (2) The Bilingual Dominance Scale (BDS; [37]) evaluates bilinguals’ life-long experience with and current use of each language (see [37], Appendix 1, for scoring). (3) Age of arrival in the United States or another English-speaking country. Table 1 displays scores on these three measures for bilinguals in each experiment separately. In Table 2 and in the remainder of this section, we describe general patterns in our language measures across all 133 bilingual participants from all 3 experiments.

**Table 1:** Basic measures of bilinguals in Experiments 1–3. Multilingual Naming Test (MINT), Bilingual Dominance Scale (BDS), and age of arrival in English-speaking country (AOA) for bilingual participants in each experiment.

*Experiment*	*Measure*	MINT Mandarin	MINT English	MINT composite	BDS	Age of arrival in years
**1**	**Mean (SD)**	50.76 (10.41)	58.98 (5.05)	-8.22 (13.56)	-2.78 (9.68)	7.18 (5.26)
**2**	**Mean (SD)**	53.42 (10.55)	55.71 (6.81)	-2.29 (14.62)	1.50 (11.44)	10.83 (6.66)
**3**	**Mean (SD)**	48.24 (12.09)	59.91 (6.78)	-11.67 (16.36)	-5.34 (11.59)	7.59 (7.11)
**Collapsed**	**Mean (SD)**	50.14 (11.28)	58.80 (6.31)	-8.65 (15.29)	-3.13 (11.06)	8.02 (6.47)
	**Range**	18–64	33–68	-47–27	-28–16	0–20

**Table 2:** Correlations between language measures. Three measures of language dominance (MINT composite, BDS, and AOA) were highly correlated with each other for the 133 Mandarin-English bilingual participants across the 3 experiments.

	**MINT Mandarin**	**MINT English**	**MINT composite**	**BDS**	**AOA**
**MINT Mandarin**		-.47***	.93***	.72***	.65***
**MINT English**			-.76***	-.68***	-.73***
**MINT composite**				.81***	.78***
**BDS**					.80***

***p < .001

For both the MINT and BDS, we subtracted the English score from the Mandarin score to compute Mandarin dominance (highly positive = strongly Mandarin dominant, and highly negative = strongly English dominant). For both measures overall, scores were slightly skewed toward English dominance (Table 1). The BDS and MINT Mandarin-dominance scores correlated strongly with each other and with age of arrival in the United States or another English-speaking country (Table 2).

## Experiment 1

Mandarin-English bilinguals and English speakers without tone-language experience learned novel words (i.e., pseudowords) containing Mandarin tonal patterns. Using novel words allowed us to manipulate the language context during learning. It also controlled listeners’ experience with the words, so that effects of Mandarin proficiency on tone use were not confounded with general “rustiness” with existing Mandarin vocabulary. Teaching novel words also enabled us to compare Mandarin-English bilinguals to monolingual English speakers.

Previous studies ([27, 35]) have taught tone distinctions to monolingual English speakers with some success. However, minimal tone pairs were used (such as /nΛk/ with rising vs. falling pitch; [27]), so success depended on attending to tones. As in Quam and Swingley’s study ([31]; see [38] for similar logic), the Experiment 1 word-set was designed so that listeners could learn words without using tone/pitch information at all. However, tone information provided an extra disambiguating cue. This tested listeners’ *spontaneous* encoding of pitch, making the experiment potentially more sensitive to effects of language experience.

For bilinguals, novel words—designed to be equally compatible with Mandarin and English in terms of their component segments—were presented in either an English or a Mandarin global language context. English speakers learned words in the English context.

### Method

#### Ethics statement

The IRB office of the University of California, San Diego (UCSD) approved this study. Participants provided written informed consent to participate.

#### Participants

*English speakers*. Twenty-six English speakers from UCSD participated (13 women, mean age, 21, *SD*, 2, range, 18–25; one age missing). Of these 26, 2 were excluded just from gaze analyses because they were fixating the pictures for less than 80% of the analyzed time-window, indicating poor eye-tracking quality. The sample included 9 bilinguals (seven Spanish-English, one Hindi-English, one Urdu-English). None had had significant experience with any tone language. Nine more participants were excluded from the analysis for the following reasons: not reaching the training criterion within the two-hour time-slot allotted (5), exposure to tone languages (2), experimenter error (1), and withdrawing from the experiment (1).

*Bilinguals*. Fifty-one Mandarin-English bilinguals participated: 25 in the Mandarin-context version (17 women, mean age, 20, *SD*, 2, range, 18–26; one age missing), and 26 in the English-context version (16 women, mean age, 20, *SD*, 2, range, 16–23; parental consent obtained when necessary). Of these 51, 3 were excluded just from gaze analyses either because they were fixating the pictures for less than 80% of the analyzed time-window, indicating poor eye-tracking quality (2), or because an experimenter error deleted gaze data (1). Fourteen additional participants were tested but replaced for the following reasons: not reaching the training criterion within the two-hour time-slot allotted (4), equivalent or greater exposure to other tone languages or dialects during childhood than to Mandarin, as indicated on post-experiment questionnaire (5), responding below 85% correct on tone-contrast trials in a familiar-word post-test (1), and experimenter error (e.g., the research assistant spoke the wrong language prior to the experiment, compromising the language-context manipulation; 4). Across all 3 experiments, all bilingual participants reported themselves as at least “fairly proficient” in both English and Mandarin, encompassing a wide range of language-dominance profiles. Most participants acquired Mandarin before English, so by a traditional definition most would be considered second-language learners of English. But by college, many of our participants had become more proficient in English than Mandarin.

#### Stimuli

Sixteen novel words (Table 3) comprised four quadruplets. Words’ segments and phonotactics were designed to be compatible with both English and Mandarin, but they contained Mandarin tones 1–4 (1 = high flat; 2 = rising; 3 = low dipping; 4 = falling) on their first syllables. Second syllables contained neutral tone. The first syllable of each word contained a consonant and vowel/diphthong that both occur in Mandarin, but never occur as a biphone, to minimize effects of syllable frequency on processing. Each word in a quadruplet (e.g., “bjoʊ3fu”) contrasted with two similar-sounding words differing in first-syllable *tone* (“bjoʊ4fa”) or *vowel* (“boʊ3fa”) as well as in second-syllable vowel. The tone vs. vowel contrast in the first syllable was designed to compare use of tone differences, with which English speakers had no experience, vs. vowel differences, with which English speakers had ample experience. The vowel contrast in the second syllable ensured that English speakers could identify the words without forcing them to exploit tone. Tone 2 and Tone 3, the pair most difficult to distinguish ([39]), were never contrasted directly.

**Table 3:** Four novel-word quadruplets. Moving horizontally within each quadruplet creates a tone contrast in the first syllable (e.g., “fi2pi” vs. “fi4pu”); moving vertically, a vowel contrast (e.g., “fi2pi” vs. “fɑʊ2pu”). Spellings here use the International Phonetic Alphabet (IPA); see https://talkbank.org/pinyin/Trad_chart_IPA.php for Pinyin equivalences.

bjoʊ3fu	bjoʊ4fa	fi2pi	fi4pu	sei1tu	sei2ti	faɪ1di	faɪ3da
boʊ3fa	boʊ4fu	fɑʊ2pu	fɑʊ4pi	swa1ti	swa2tu	fjɑʊ1da	fjɑʊ3di

Auditory stimuli were recorded in a sound-attenuated chamber and normalized to a mean amplitude of 70 decibels in Praat ([40]). Sentences were recorded by a native Mandarin speaker who was born in Taiwan, and began learning English at age seven. The speaker was slightly English dominant according to both language assessments described above, with a MINT dominance score of -4 and BDS score of -3. We sought out a relatively balanced bilingual speaker rather than a Mandarin monolingual or Mandarin-dominant bilingual so that they could record both Mandarin and English carrier phrases for the language-context manipulation. A control experiment (described in Experiment A in S1 Supplemental Materials) verified that our speaker’s Mandarin accent on these stimuli was equivalent to a very Mandarin-dominant speaker from mainland China.

Each target word was recorded once in an English carrier phrase (“Choose the [boʊ4fa].”) and twice in a Mandarin carrier (“Qing3 xuan3 [boʊ4fa].”). The best token from one of the Mandarin recordings (judged by the bilingual speaker and the authors to have the best tone contour and overall recording quality) was spliced into the English carrier and into the second Mandarin carrier. Two additional native-Mandarin speakers verified tone pronunciations. In response to their observation that Tones 2 and 3 sounded very similar, we used Praat *Pitch Resynthesis* to resynthesize the pitch contours of Tone 2s to rise more linearly (vs. dipping slightly, which made them confusable with Tone 3).

#### Apparatus and procedure

All participants first completed the novel-word-learning experiment. In the Mandarin context, pre-experiment instructions were in Mandarin, and stimulus sentences were “Qing3 xuan3 [boʊ4fa]” (“Please choose [boʊ4fa]”). In the English context, all instructions and stimulus sentences (“Choose the [boʊ4fa]”) were in English. The experiment contained training and test phases. On each training and test trial, two pictures at a time appeared on the screen. The objects were unfamiliar black-and-white shapes, drawn from a set of shapes used in several prior experiments (e.g., [38, 41–43]). Pictures were 150 X 150 pixels, centered at 23% and 73% of screen width, respectively, and 47.6% of height (deviating slightly from 25%, 75%, and 50% due to a minor programming miscalculation). After 500 milliseconds, a sentence, played over Sennheiser HD 280 pro headphones, labeled one of the pictures. Participants clicked the computer mouse on the picture they thought matched the last word in the sentence, guessing if necessary.

During training, the two pictures on the screen had names from distinct quadruplets (see Table 3), which contained different tones whenever possible, to make learning easier and reduce the likelihood that participants would detect the experimental manipulation. Each picture was the target 8 times per 128-trial block, and appeared equally often as the competitor. During training, participants received feedback: after they clicked a picture, the correct picture stayed on the screen while the incorrect picture disappeared. A Matlab script written using the PsychToolbox3 ([44, 45]) tabulated accuracy in each block. Once a participant reached 90% accuracy within a training block, they proceeded to the test phase. Training time did not differ significantly between groups (bilinguals, Mandarin-context: *M*, 2.28 blocks, *SD*, 0.94; bilinguals, English-context: *M*, 2.58 blocks, *SD*, 1.03; English speakers: *M*, 2.5 blocks, *SD*, 0.91).

Test trials were similar to training trials, but lacked response feedback. Half of the 128 trials were *baseline* trials, comparable to training trials in that the two pictures had very distinct names. In the other half of test trials, word pairs were taken from the same quadruplet, differing either in their *vowels* (e.g., “fi2pi” vs. “faʊ2pu”), or in their *tones* (e.g., “fi2pi” vs. “fi4pu”). Each word occurred equally often as target vs. competitor within each trial type, so that word frequencies did not provide cues to the correct response. Accuracy and visual fixations to each picture were measured. An Eyelink Remote eye-tracker (SR Research; www.sr-research.com) collected visual fixation data using the Eyelink toolbox for Matlab ([46]). Following the test phase, bilinguals completed a Mandarin familiar-word recognition experiment with tone and vowel minimal pairs (details reported in [47]), followed by the MINT and BDS.

### Results

We collected clicking accuracy and eye-gaze data as indices of participants’ learning. Accuracy data told us about participants’ decisions, whereas eye movements provided information about participants’ fine-grained temporal processing. Because the proportion of error responses was already reflected in the accuracy data, we excluded error trials from gaze analyses so that the two measures would be more independent. Gaze analyses on correct trials alone might reveal processing differences even when participants ultimately clicked on the correct picture (e.g., [47, 48]).

To statistically compare responses, we computed the empirical-logistic (e-logit) transform on accuracy as well as on gaze ([49]). For gaze, target fixations and competitor fixations were each e-logit transformed; we then analyzed the target—competitor subtraction, or “target advantage,” for these transformed values. Raw means are reported in tables and figures for ease of interpretability. As words in baseline trials were paired to be maximally dissimilar, these trials were not sensitive to differences across groups (see Table 4), so were not analyzed further.

**Table 4:** Means (standard deviations in parentheses) for accuracy across language-context groups (with English speakers included for comparison) and trial types in Experiment 1.

	Bilinguals			English speakers
Trial type	Mandarin context	English context	All	
**Baseline trials**	98.8% (1.4%)	98.7% (2.2%)	98.7% (1.8%)	98.1% (2.6%)
**Different-vowel trials**	93.5% (6.3%)	94.1% (6.6%)	93.8% (6.4%)	90.1% (8.2%)
**Different-tone trials**	83.0% (12.2%)	85.6% (10.0%)	84.3% (11.1%)	79.6% (10.3%)
**All trials**	91.8% (10.3%)	92.8% (8.9%)	92.3% (9.6%)	89.3% (10.8%)

#### Comparing bilinguals’ vs. English speakers’ accuracy and gaze

We first asked how bilinguals’ overall accuracy compared to English speakers. ANOVAs on accuracy contained Trial Type (different-vowel vs. different-tone) and Language Group (bilinguals vs. English speakers) as factors. A main effect of Trial Type (F1(1,75) = 81.86, *p* < .001; F2(1,15) = 57.82, *p* < .001), showed greater overall accuracy for vowels (*M*, 92.6%, *SD*, 7.0%) than tones (*M*, 82.7%, *SD*, 11.0%). Results from a separate gating experiment using the Experiment 1 stimuli (described in Experiment C in S1 Supplemental Materials) could explain this result. In the gating experiment, 12 additional bilingual participants were able to disambiguate vowel contrasts earlier in the signal than tone contrasts.

A main effect of Language Group (F(1,75) = 4.98, *p* < .05; F2(1,15) = 5.78, *p* < .05), indicated greater accuracy (across vowel and tone trials collapsed) for bilinguals (*M*, 89.1%, *SD*, 7.8%) than English speakers (*M*, 84.9%, *SD*, 8.0%). The two factors did not interact. However, because of our *a priori* interest in each group’s tone processing specifically, we conducted planned comparisons between the two language groups for each trial type separately. Bilinguals were marginally more accurate than English speakers in vowel trials (t1(75) = 1.84, *p* = .07; t2(15) = 2.06, *p* = .057), and more accurate (by-subjects) in tone trials (t1(75) = 2.06, *p* < .05; t2(15) = 1.87, *p* = .082).

We next asked how bilinguals’ overall gaze patterns compared to English speakers. ANOVAs on gaze revealed analogous patterns to the accuracy ANOVA. There was a main effect of Trial Type (F1(1,70) = 23.15, *p* < .001; F2(1,15) = 34.53, *p* < .001), with greater target-advantage scores for vowels than tones (see Table 5 for means). There was also a main effect of Language Group (F(1,70) = 8.05, *p* < .01; F2(1,15) = 18.01, *p* = .001), indicating greater target-advantage scores for bilinguals than English speakers. The two factors did not interact. Again, because of our *a priori* interest in each group’s tone processing specifically, we conducted planned comparisons between the two language groups for each trial type separately. Bilinguals had significantly higher target-fixation scores than English speakers in both vowel trials (t1(70) = 2.24, *p* < .05; t2(30) = 2.09, *p* < .05) and tone trials (t1(70) = 2.42, *p* < .05; t2(30) = 2.33, *p* < .05).

**Table 5:** Means (standard deviations in parentheses) for target advantage across language-context groups (with English speakers included for comparison) and trial types in Experiment 1.

	Bilinguals			English speakers
Trial type	Mandarin context	English context	All	
**Baseline trials**	0.42 (0.13)	0.41 (0.12)	0.42 (0.12)	0.39 (0.11)
**Different-vowel trials**	0.33 (0.14)	0.33 (0.09)	0.33 (0.12)	0.26 (0.13)
**Different-tone trials**	0.27 (0.12)	0.23 (0.12)	0.25 (0.12)	0.18 (0.11)
**All trials**	0.34 (0.14)	0.32 (0.13)	0.33 (0.14)	0.27 (0.15)

#### Effects of language context and language dominance on bilinguals’ accuracy and gaze

**Fig 1:**
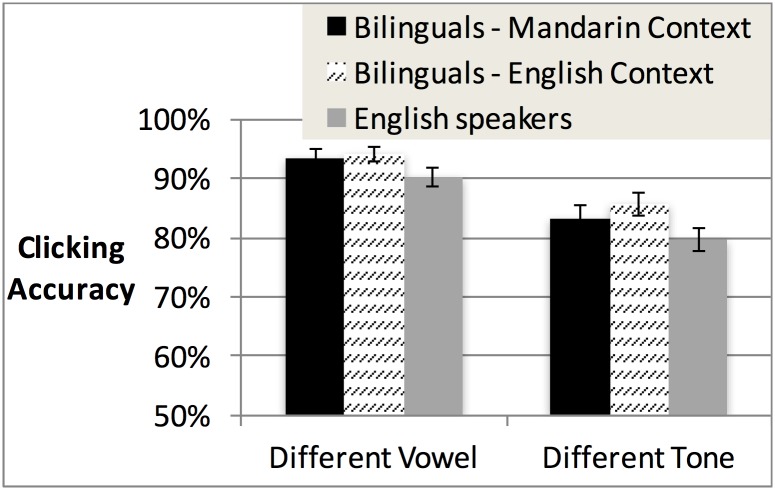
Raw accuracy scores in different-vowel vs. different-tone trials in Experiment 1, for bilinguals in the Mandarin context vs. the English context. English speakers are included for comparison.

We next considered whether the language context and degree of Mandarin dominance affected bilinguals’ accuracy. Initial analyses only considered the presence or absence of Mandarin knowledge (comparing bilinguals vs. English speakers), but gradient effects of degree of dominance in Mandarin could also impact accuracy. Analyses of covariance (ANCOVAs) were conducted with Trial Type (vowel vs. tone) and Language Context (Mandarin vs. English) as categorical predictors and Mandarin dominance on the MINT vocabulary test as a continuous covariate. The ANCOVA revealed a main effect of Trial Type (F(1,48) = 41.18, *p* < .001), with higher accuracy for different-vowel than different-tone trials (see Fig 1). There was also a main effect of the Mandarin dominance covariate (F(1,48) = 6.88, *p* < .05). No other effects or interactions were significant. Because of our a priori interest in the impact of language dominance on tone processing specifically, we conducted Pearson’s correlation tests in each trial type separately. These revealed that bilinguals showed better accuracy for both different-vowel (*r* = .28, *p* < .05) and different-tone trials (*r* = .34, *p* < .05) as Mandarin dominance increased. The lack of Language Context effects or interactions indicates that the experiment-wide language context did not alter bilinguals’ processing of tone information.

**Fig 2:**
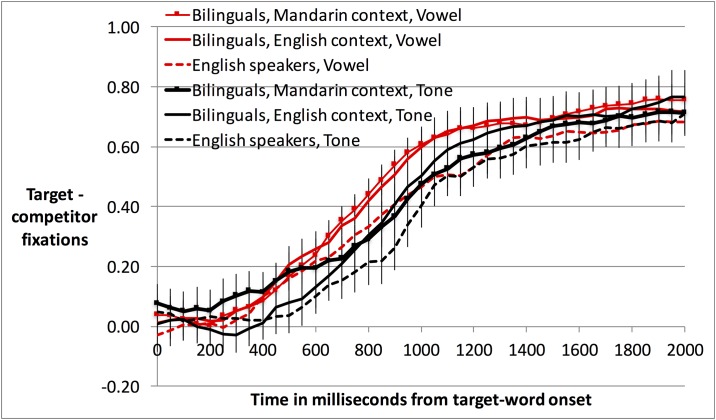
Eye-gaze target-advantage in Experiment 1. Throughout, error bars are standard errors.

We conducted an analogous ANCOVA on gaze patterns. Fig 2 depicts participants’ eye-gaze responses across time as raw target minus competitor fixations, or “target advantage.” This number ranges from roughly 0, chance looking, to 1, looking only at the target. Trial lengths were variable, ending when participants clicked on a picture, so for trials that ended prior to 2000 ms post-word, we extended the final fixation of each trial to 2000 ms so that all trials contributed equally across the full time course in figures and analyses. For analysis, we then averaged e-logit-transformed target advantage across the time window 200–1100 ms after the onset of the target word. The start of this window represents the earliest point at which adults can initiate an eye-movement response ([50]). The end of this window, 1100 ms, was selected to balance two opposing factors: best reflecting the overall asymptote in Fig 2, while keeping the percentage of extended bins within the analyzed time window as low as possible (6.9% in Experiment 1 across different-vowel and different-tone trials, 7.2% in Experiment 2, and 7.5% in Experiment 3).

As in the accuracy ANCOVA, the gaze ANCOVA revealed a main effect of Trial Type (F(1,45) = 14.35, *p* < .001), indicating higher target advantage in different-vowel than different-tone trials (see Table 5 for means and Fig 2 for timecourse plot). There was also a main effect of the Mandarin dominance covariate (F(1,45) = 4.24, *p* < .05). Given our a priori interest in tone processing specifically, we conducted Pearson’s correlation tests between target advantage and Mandarin dominance within each trial type. These correlation tests showed a different pattern than the accuracy results: target advantage numerically *decreased* for both different-vowel (r = -.22, n.s.) and different-tone trials (r = -.27, *p* = .067) as Mandarin dominance increased. Again, there were no effects of Language Context, indicating that there was no learning advantage for bilingual participants who had been consented, trained, and tested in a Mandarin environment.

### Discussion

Experiment 1 yielded two major findings. First, success in learning and recognizing words with tones was related to experience with Mandarin. Mandarin-English bilinguals were both more accurate word learners and more efficient at visually identifying the referents of novel words than English speakers without tone experience. For bilinguals, accuracy overall was also correlated with Mandarin dominance. For gaze, surprisingly, the correlation between target advantage and Mandarin dominance was in the opposite direction. One speculation is that this could be due to a speed-accuracy trade-off. Given that the less Mandarin-dominant bilinguals made more errors, a larger proportion of the “correct” trials for the less Mandarin-dominant bilinguals may have actually been lucky guesses. It could be that when participants are guessing, they identify the target picture more rapidly than when they are carefully selecting the right answer. Of course, correlations within each trial type were non-significant by-subjects, suggesting the possibility that the Mandarin-dominance effect was a false positive.

The second major result was the *absence* of an effect of global language context on bilinguals’ processing. Conducting the experiment in Mandarin as opposed to English did not result in higher accuracy or target advantage, on different-tone trials or elsewhere. Why not? One possibility is that our listeners were unable to adjust their processing strategies to the context, and instead processed all spoken words through whichever phonological system was dominant. A second possibility is that listeners *are* able to adjust processing to the context, but the relevant language context is the phonetic/phonological content of the word itself. Recall that we carefully controlled segmental content to provide no cues *within the word itself* as to the language membership of the word. However, listeners may be capable of adjusting to context on a word-by-word basis, if within-word cues are available.

Supporting the second possibility, we found evidence that the stimuli themselves, despite our careful balancing of segmental content, may have been *overall* more Mandarin-like phonetically. Control experiments (described in S1 Supplemental Materials, *Experiments A* and *B*) suggested that our Experiment 1 speaker was identifiable as Chinese, even when she was producing target words in English carrier phrases (e.g., “Choose the biu3fu”; Experiment A in S1 Supplemental Materials). However, the words themselves were, as designed, fairly well balanced between Mandarin and English phonemes and phonotactics (Experiment B in S1 Supplemental Materials). Still, it may be that the presence of Mandarin tones and the speaker’s identifiability as Chinese—presumably based on subphonemic accentual cues—may have biased bilinguals to encode the words using their Mandarin phonetic system regardless of the language of the instructions and carrier phrases. That is, we may have triggered a *phonetic-*context effect that was stronger than the language-context effect. This could explain why we found bilingual accuracy advantages for both tones and vowels.

In the next experiment, we explored the other half of the phonetic-context effect: would bilinguals’ advantage in learning tone-bearing words disappear if tone were embedded in words that sounded English-like? The advantages we saw for tone (and vowel) use among Mandarin-dominant bilinguals in the first experiment could indicate either overall greater attention to tone (or even that bilinguals are simply better learners [51]; though see [52] for a different perspective), or it could indicate greater encoding of words with tone information *specifically in a Mandarin-like context*. The second experiment differentiated these explanations by teaching tone-bearing English-like words, that is, an American-English-accented talker producing strongly phonologically English-biased words that nonetheless had Mandarin tonal patterns. If bilinguals can modulate their use of tone to match the language context, they should attend less to tone in this case, and their tone use should be no better than English speakers’. By contrast, if listeners simply process sounds through the filter of their dominant language, irrespective of the language context, then results for each language group should mirror Experiment 1.

## Experiment 2

### Method

#### Participants

*English speakers*. Twenty-five English speakers participated (19 women, mean age = 21, *SD*, 2, range = 18–25), including one Spanish-English bilingual. Of these 25, 1 was excluded just from gaze analyses for looking less than 80% at the pictures during the analyzed time window, indicating poor eye-tracking quality. One additional participant was tested but excluded from all analyses for a computer error (1).

*Bilinguals*. Twenty-four bilinguals participated (17 women, mean age = 20, *SD*, 2, range = 18–23). Of these 24, 1 was excluded just from gaze analyses for looking less than 80% at the pictures during the analyzed time window. This sample was roughly half the size of the bilingual sample in Experiment 1 because there was no between-subjects manipulation. Inclusion criteria matched Experiment 1. Eight additional participants were tested but replaced for the following reasons: failing to complete the experiment within the allotted two-hour time slot (6); equivalent or greater exposure to other tone languages or dialects during childhood than to Mandarin (2).

#### Stimuli

Sixteen words (see Table 6) contained the same tone patterns as the novel words from Experiment 1, but had very English-like phonetic/phonological content: they were pronounced by a native English speaker, included vowels only occurring in English, and included consonant clusters, which do not occur in Mandarin. They were recorded by an American-English speaker (CQ, from Washington State), a musician with extensive training in pitch modulation. A Mandarin-English bilingual selected tokens with the most accurate tone patterns. A control experiment (see Experiment D in S1 Supplemental Materials) verified that tones were equally identifiable for these stimuli as for those in Experiment 1.

**Table 6:** Four English-like novel-word quadruplets. Stimulus design is akin to **Experiment 1,** but consonants and vowels are strongly English-like. Spellings use IPA.

klæ3fu	klæ4fa	gri2pi	gri4pu	plæ1tu	plæ2ti	bræ1di	bræ3da
kl∧3fa	kl∧4fu	gr∧2pu	gr∧4pi	pli1ti	pli2tu	bri1da	bri3di

#### Apparatus and procedure

The experiment was analogous to Experiment 1, English context. Training duration did not differ significantly between groups (bilinguals: 2.46 blocks, *SD*, 0.66; English speakers: 2.24 blocks, *SD*, 0.78).

### Results

We again analyzed both accuracy and gaze responses, and included only different-vowel and different-tone trials in statistical analyses, following the same procedures as in Experiment 1.

#### Comparing bilinguals’ vs. English speakers’ accuracy and gaze

**Fig 3:**
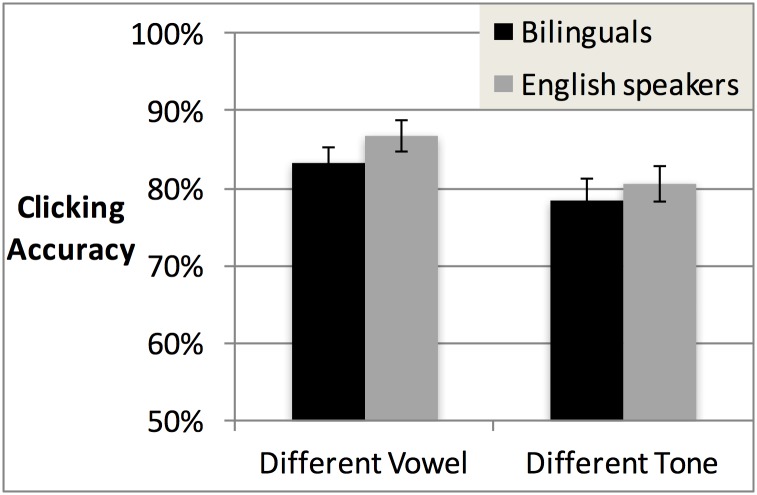
Accuracy scores for bilinguals vs. English speakers in Experiment 2, in different-vowel vs. different-tone trials.

ANOVAs on accuracy were conducted with Trial Type (different-vowel vs. different-tone) and Language Group (bilinguals vs. English speakers) as factors. Only Trial Type was significant (F1(1,47) = 17.83, *p* < .001; F2(1,15) = 13.08, *p* < .005), with higher accuracy for different-vowel than different-tone trials (see Table 7 and Fig 3 for means). Neither Language Background (F1(1,47) = 1.10, *p* = 0.96; F2(1,15) = 3.87, *p* = .068) nor the Language Background x Trial Type interaction (F1(1,47) = 1.33, *p* = 0.26; F2(1,15) = 1.38, *p* = .258) reached significance, indicating that bilinguals performed similarly to English speakers.

**Table 7:** Means (SDs) for accuracy across language groups and trial types in Experiment 2.

	Bilinguals	English speakers	Overall
**Baseline trials**	99.1% (1.1%)	98.5% (3.0%)	98.8% (2.3%)
**Different-vowel trials**	83.2% (9.9%)	86.8% (9.5%)	85.0% (9.8%)
**Different-tone trials**	78.4% (13.6%)	80.5% (11.2%)	79.5% (12.4%)
**All trials**	86.9% (13.1%)	88.6% (11.4%)	87.8% (12.2%)

**Fig 4:**
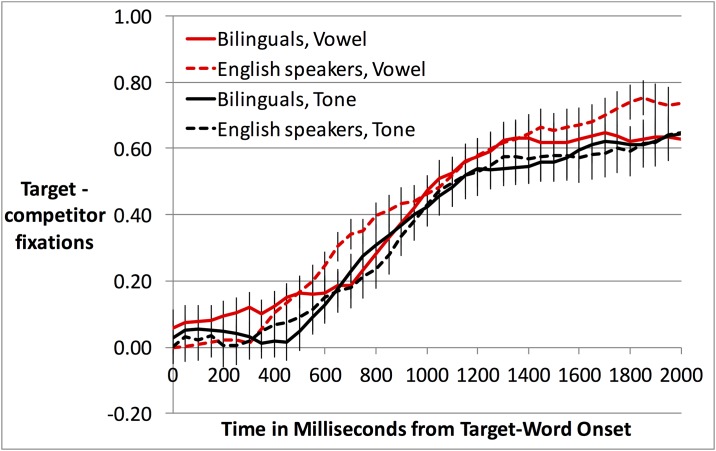
Target advantage over time for English-like novel words by language group. Baseline trials not pictured.

For a similar ANOVA on gaze patterns (Fig 4, Table 8), there was again a main effect of Trial Type (F1(1,45) = 5.28, *p* < .05; F2(1,15) = 8.66, *p* = .01), reflecting higher target-advantage scores in different-vowel than different-tone trials. Also like the accuracy data, no other effects approached significance.

**Table 8:** Means (SDs) for target advantage across language groups and trial types in Experiment 2.

	Bilinguals	English speakers	Overall
**Baseline trials**	0.40 (0.11)	0.47 (0.16)	0.44 (0.14)
**Different-vowel trials**	0.26 (0.15)	0.28 (0.15)	0.27 (0.15)
**Different-tone trials**	0.21 (0.15)	0.21 (0.16)	0.21 (0.16)
**All trials**	0.29 (0.16)	0.32 (0.19)	0.31 (0.18)

#### Effects of language dominance on bilinguals’ accuracy and gaze

As in Experiment 1, to address whether tone processing in bilinguals varied with proficiency in Mandarin, two ANCOVAs evaluated differences in accuracy and gaze, respectively, with Trial Type and (continuous) Mandarin Dominance as factors. Unlike in Experiment 1, for both accuracy and gaze, we found no effects of or interactions with Mandarin dominance (n.s.).

#### Cross-experiment effects of phonetic/phonological context on accuracy and gaze

To consider the effects of the phonetic content of the target words, which differed between Experiments 1 and 2, we conducted cross-experiment analyses. First, we compared bilinguals vs. English speakers across experiments. ANOVAs on accuracy included factors Trial Type, Experiment (between-subjects, between-items), and Language Background (bilinguals vs. English speakers; between-subjects, within-items). There were significant main effects of Trial Type (F1(1,122) = 81.86, *p* < .001; F2(1,30) = 64.56, *p* < .001), with different-vowel trials more accurate than different-tone trials, and Experiment (F1(1,122) = 6.77, *p* = .01; F2(1,30) = 8.69, *p* < .01), with lower overall accuracy in Experiment 2 than Experiment 1. Trial Type and Experiment also interacted (F1(1,122) = 14.33, *p* < .001; F2(1,30) = 9.71, *p* < .005), indicating that lower accuracy in Experiment 2 vs. Experiment 1 was driven mainly by different-vowel trials (t1(101.19) = 4.22, *p* < .001; t2(30) = 4.10, *p* < .001), though different-tone trials showed a numerical effect in the same direction. Note that, by design, words were very different between the two experiments, so a number of factors could have generated accuracy differences in vowel trials. One possibility is that the greater proportion of vowels shared across words in Experiment 2—a result of adhering to a limited set of vowels incompatible with Mandarin when creating the English-compatible words—could have made vowel contrasts more confusable overall. (Experiment 3, below, uses more closely matched sets of Mandarin-like vs. English-like words.)

The interaction of Experiment with Language Group was also significant (F1(1,122) = 4.71, *p* < .05; F2(1,30) = 9.64, *p* < .005). This was because bilinguals showed significantly *higher* overall accuracy in Experiment 1 than English speakers (t1(55.80) = 2.32, *p* < .05; paired t2(15) = 2.40, *p* < .05), but the two groups did not differ significantly in Experiment 2 (t’s < 1).

As in accuracy analyses, ANOVAs evaluated gaze with factors Trial Type, Experiment, and Language Background. There was a significant main effect of Trial Type (F1(1,115) = 23.05, *p* < .001; F2(1,30) = 34.97, *p* < .001), again indicating higher target advantage for vowels than tones. There was also a marginal interaction of Experiment and Language Group (F1(1,115) = 3.70, *p* = .057; F2(1,30) = 4.80, *p* < .05), indicating that bilinguals showed significantly *higher* overall target advantage in Experiment 1 than English speakers (t1(47.64) = 2.87, *p* < .01; paired t2(15) = 4.24, *p* = .001), but the two groups did not differ significantly in Experiment 2 (t’s < 1).

The results of these two ANOVAs are consistent with bilinguals showing an advantage over English speakers for learning Mandarin-like words (Experiment 1), but not words with English-like phonetics and phonology (Experiment 2).

### Discussion

In the current experiment, listeners learned novel words that were strongly biased toward English phonology and phonotactics. When recognizing these words, bilinguals’ use of vowel and tone information did not differ from English speakers’. Cross-experiment analyses revealed that this pattern contrasted statistically with Experiment 1, where bilinguals’ accuracy and (visual) efficiency at exploiting both vowel and tone information surpassed English speakers’.

The current experiment helps address the question of whether bilinguals’ word-learning advantage in Experiment 1 reflected a general “bilingual advantage” in word learning ([51]). If bilinguals were simply superior overall at word learning, they should have exceeded English speakers’ accuracy not only in Experiment 1, but in the current experiment as well—yet they did not. Thus, the current experiment is more consistent with the account that bilinguals match their word encoding to the within-word phonological/phonetic context, encoding tone more strongly than other groups only when the within-word context matches Mandarin.

While a comparison between Experiments 1 and 2 suggests that bilinguals attend more to the same tone contours in Mandarin-like words than in English-like words, it was not clear whether this pattern was limited to tones, or was true of all Mandarin-like stimuli, given that we saw comparable Mandarin-speaker advantages in different-vowel trials as well. A *within-subjects* comparison, with more closely matched sets of Mandarin-like and English-like words, would provide an even stronger test of whether bilinguals can match their tone processing to the within-word language context. This was pursued in Experiment 3.

## Experiment 3

Experiment 3 provided a stronger test of whether bilinguals can process tone language-specifically, by manipulating language context within subjects. We taught each participant two sets of words, one Mandarin-like and the other English-like. Experiment 3 also equated word characteristics across the two word sets as much as possible, in order to determine whether bilinguals would still process vowels—not just tones—more efficiently in Mandarin than in English when very similar vowels were used in both word sets.

We also sought to determine whether language background would still impact tone use even when tone content was more noticeable. Unlike Experiments 1 and 2, we used a word set and training paradigm more similar to previous studies ([27, 35]) that taught tone words to English speakers by providing feedback on minimal tone pairs. While Experiments 1 and 2 tested listeners’ inclinations to use tone when their attention was not specifically drawn to it, here we were able to ask how *capable* different groups of listeners were of attending to tone when tone content was highlighted. If English speakers still process tone less efficiently than Mandarin-English bilinguals despite having their attention directed to tonal features, this will provide even stronger evidence that experience with Mandarin influences the *ability* (not just the proclivity) to exploit lexical-tone content in word learning.

We made tone content more noticeable in three ways. First, we explained to all participants that some words they would learn might differ only in their pitch patterns. Instructions were carefully designed to be comprehensible to people with no experience with tone languages:

“For the words you are going to learn, tone (pitch, or the height of the voice) is going to be important for telling apart some of the words. So you should try to pay attention to whether the voice is high or low, and how the pitch is rising or falling. An example from English would be how your pitch is different when you ask a question ("Do you see that car?") vs. say a statement ("Look at that car."). Another example is that kids tend to have higher-pitched voices than adults, and women’s voices tend to be higher than men’s. This is the way that these words might differ from each other, so it will be useful to pay attention to the pitch.”

Second, each word in the set (e.g., dei2) had a sister word contrasting only in tone (dei4). Experiment 1 and 2 words had had an additional disambiguating element besides tone—the vowel in the 2nd syllable. Third, 1/7 of training trials used two pictures whose labels were minimal tone pairs (dei2 and dei4 presented side-by-side). Thus, attending to tone was necessary to respond accurately in those trials, and participants received feedback in those trials on their ability to distinguish minimal tone pairs. In Experiments 1 and 2, different-tone pairs had never been paired during training.

### Method

#### Participants

*English speakers*. Twenty-eight English speakers participated (15 women, mean age = 20, *SD*, 2, range = 18–25), including one Tagalog-English bilingual and one Gujarati-English bilingual. One of the 28 was excluded only from gaze analyses because of target plus competitor fixations of below two-thirds, or 67%, during the analyzed time window. (Note that a less stringent fixation criterion was employed here vs. Experiments 1 and 2 because the lack of a carrier phrase in the auditory stimuli meant that at target-word onset, more participants were still fixating the central fixation point.) Seven additional participants were tested but replaced due to: not completing the experiment within the allotted time (3), significant exposure to a pitch-accent language (2), being an age outlier (> 10 SDs above the mean); 1), and wearing hearing tubes (1).

*Bilinguals*. Fifty-eight bilinguals participated (37 women; mean age = 20, *SD*, 1, range = 18–23). We roughly doubled the number of bilinguals relative to English speakers in this experiment to facilitate assessment of Mandarin-dominance effects for bilinguals. Inclusion criteria matched Experiment 1. Six bilinguals were excluded only from gaze analyses because of experimenter error (4) or target plus competitor fixations of below two-thirds, or 67%, during the analyzed time window (2). Seven additional participants were excluded from all analyses for the following reasons: not completing the experiment within the allotted time (4), not being a native speaker of Mandarin (1), having equivalent or greater exposure to other tone languages or dialects during childhood than to Mandarin (1), and responding below 85% correct on tone-contrast trials in a familiar-word post-test (1).

#### Stimuli

Sixteen novel words comprised two wordsets (see Table 9). Each wordset contained all 4 tones across the 8 words. As in previous experiments, each word had a tone competitor and a vowel competitor, but here, because words were monosyllabic, different-tone pairs differed only in tone (and different-vowel pairs differed only in vowel). In order to contrast the within-word language context, each word had an English-like and a Mandarin-like variant. Mandarin words had the consonant-vowel (CV) structure typical of Mandarin syllables (e.g., dei). The English words differed from the Mandarin words in that they contained coda consonants not sanctioned by Mandarin phonotactics (e.g., deish)—however, final consonants were identical within each tone- and vowel-disambiguated word pair. Thus, unlike the wordsets in Experiment 1 vs. Experiment 2, the Mandarin and English wordsets used here contained the same (or very similar) vowel categories, though they were pronounced with subphonemic/accentual information consistent with each language. The tone content was equated across the two wordsets using pitch resynthesis (see below). As carrier phrase language had no effect in Experiment 1, words were presented in isolation rather than in carrier phrases.

**Table 9:** Novel-word sets in Experiment 3. Numbers indicate Mandarin tones 1–4. Spellings are in IPA. For each word in each set, there was a Mandarin-like variant and an English-like variant (transcribed below as Mandarin/English). Each participant was taught both wordsets, but heard the English variants of one wordset (e.g., fjɑʊd3) and the Mandarin variants of the other wordset (e.g., dei2).

	Set 1		Set 2	
*Mandarin-like words*	fjɑʊ3 fjɑʊ4	nei1 nei2	dei2 dei4	mɤ1 mɤ3
	fo3 fo4	noʊ1 noʊ2	dja2 dja4	mju1 mju3
*English-like words*	fjɑʊd3 fjɑʊd4	neid⌢ʒ1 neid⌢ʒ2	deiʃ2 deiʃ4	m^b1 m^b3
	foʊd3 foʊd4	noʊd⌢ʒ1 noʊd⌢ʒ2	djaʃ2 djaʃ4	mjub1 mjub3

The Mandarin words were recorded by a native speaker of Mandarin from Hsinchu, Taiwan who was attending graduate school in the US and was a Mandarin-dominant bilingual. Each Mandarin-like token was recorded twice to allow tone splicing as described below. English words were recorded by the same native speaker of American English from Experiment 2, who attempted to match Mandarin tokens for pitch and duration, so as to facilitate pitch resynthesis of tone contours from the Mandarin stimuli. Tone contours from one set of Mandarin novel-word tokens were extracted and carefully superimposed onto different tokens of the Mandarin and English words using Praat software ([40]). Each participant heard the English version of one wordset and the Mandarin version of the other. The pairing of language and wordset was counterbalanced across participants.

#### Apparatus and procedure

A similar methodology to the previous experiments was employed, in which participants first completed the novel-word-learning experiment, then the familiar-word recognition experiment, then assessments of language background and/or dominance. The primary change from previous experiments was that the novel-word-learning experiment consisted of *two* consecutive word-learning+test phases, one for each language context (order counterbalanced across participants).

The 112 trials in each word-learning training block consisted of 64 “baseline” trials (4/7 of the total), in which pictures were paired so that their labels were maximally distinct, and 16 trials each (1/7 of the total) of different-vowel trials (e.g., nei(g)1 vs. nou(g)1), different-tone trials (nei(g)1 vs. nei(g)2), and different-tone&vowel trials (nei(g)1 vs. nou(g)2). The test phase for each language context consisted of 64 trials, which were equally divided between the same 4 trial-types. Each set of 16 tone-, vowel-, and different-tone&vowel trials (for both training and test) contained each of the 8 words presented twice as the target and twice as the competitor.

Participants completed a maximum of 3 training blocks per language context, moving to the test phase once they had reached 90% correct responses. Training duration did not differ significantly between groups (bilinguals: 1.64 blocks, *SD*, 0.65; English speakers: 1.64 blocks, *SD*, 0.59), but was significantly longer overall for the English context than for the Mandarin context (English: 1.76 blocks, *SD*, 0.66; Mandarin: 1.51 blocks, *SD*, 0.57; t(85) = 2.77, *p* < .01; both groups showed this numerical pattern). All instructions were presented in English.

### Results

As before, we considered accuracy and gaze responses. To match previous experiments, only different-vowel and different-tone trials were entered into statistical analyses. The order in which participants completed the Mandarin vs. the English training did not interact with the language group, so, for simplicity, it was not included as a factor in the ANOVAs (though see **Footnote 2**).

#### Effects of phonetic/phonological context on bilinguals’ vs. English speakers’ accuracy and gaze

Since we trained all participants to criterion on trials including the critical contrasts (i.e., minimal tone and minimal vowel pairs), this may have muted accuracy differences at test. Nonetheless, we assessed the impact of the language context on each language group (bilinguals and English speakers). We first conducted a preliminary by-subjects ANOVA with factors Trial Type (different-vowel vs. different-tone), Language Context (Mandarin-like words vs. English-like words; within-subjects and within-items; e.g., nei1 and neid⌢ʒ1 were treated as the same item in statistical tests), Group (bilinguals vs. English speakers), and the additional between-subjects factor of Language Order (Mandarin training first vs. English training first), with the latter factor included in order to ascertain whether differences between bilinguals and English speakers might have been suppressed by learning English-like words before Mandarin-like words. There were no effects of Language Order, so the main ANOVAs reported below collapse across Language Order for simplicity, including only factors Trial Type, Language Context, and Group.

ANOVAs were conducted on accuracy with factors Trial Type, Language Context, and Group. The ANOVAs revealed a significant main effect of Trial Type (F1(1,84) = 11.38, *p* = .001; F2(1,15) = 10.66, *p* < .01), indicating higher accuracy overall in vowel trials (*M*, 93.8%, *SD*, 8.8%) than tone trials (*M*, 90.6%, *SD*, 10.7%). There was also a main effect of Group (F1(1,84) = 4.92, *p* < .05; F2(1,15) = 5.08, *p* < .05), indicating overall higher accuracy for bilinguals (*M*, 92.9%, *SD*, 9.9%) than English speakers (*M*, 90.7%, *SD*, 9.6%), as in Experiment 1. No other effects approached significance by both subjects and items.

**Fig 5:**
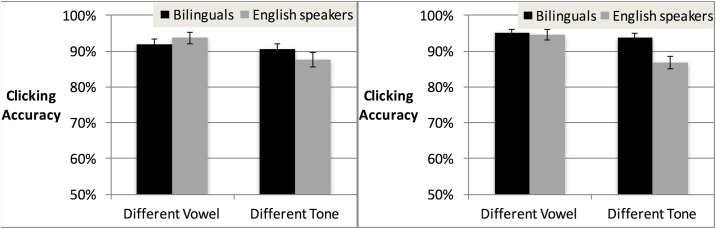
Accuracy for bilinguals and English speakers in each trial type in the English context (left) and Mandarin context (right) of Experiment 3.

Because of our a priori prediction that bilinguals would be more accurate than English speakers in tone trials in the Mandarin context specifically, we conducted separate ANOVAs for each trial type, with factors Language Context and Group. The ANOVA for vowel trials revealed no significant effects. By contrast, the ANOVA for tone trials revealed a significant main effect of Group (F1(1,84) = 9.84, *p* < .005; F2(1,15) = 14.7, *p* < .005), indicating higher accuracy among bilinguals (*M*, 92.2%, *SD*, 9.1%) than English speakers (*M*, 87.2%, *SD*, 7.7%). The interaction of Group and Language Context did not reach significance. This might be interpreted as an *overall* bilingual advantage for tone. Nevertheless, given our interest in tone processing in the Mandarin vs. the English context, we conducted follow-up comparisons, which revealed that bilinguals’ tone accuracy significantly exceeded English speakers’ in the Mandarin context (t1(84) = 3.78, *p* < .001; t2(15) = 3.46, *p* < .005, significant after Bonferroni correction) but not in the English context (n.s.; see Table 10 and Fig 5 for means).

**Table 10:** Means (SDs) for accuracy across language groups and trial types in Experiment 3.

	English context		Mandarin context	
	Bilinguals	English speakers	Bilinguals	English speakers
**Baseline trials**	99.4% (2.2%)	99.8% (1.2%)	99.8% (1.2%)	100.0% (0.0%)
**Different-tone&vowel trials**	98.6% (3.9%)	98.4% (3.7%)	98.8% (4.1%)	99.3% (2.0%)
**Different-vowel trials**	91.9% (10.3%)	93.8% (8.5%)	95.2% (7.4%)	94.6% (7.9%)
**Different-tone trials**	90.6% (11.4%)	87.5% (10.6%)	93.8% (9.9%)	86.8% (8.9%)
**All trials**	95.1% (8.9%)	94.9% (8.5%)	96.9% (7.0%)	95.2% (8.0%)

**Fig 6:**
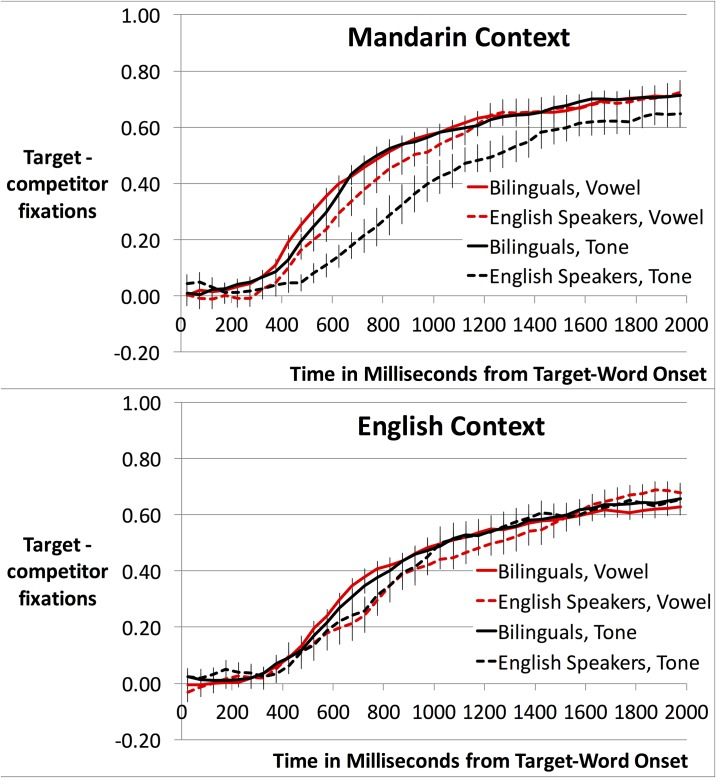
Target advantage over time for bilinguals and English speakers in Experiment 3, in the Mandarin context (top) and the English context (bottom), with error trials excluded.

Whereas training to criterion on minimal pairs may have muted accuracy differences as a function of our variables of interest, gaze patterns might be more sensitive. Target advantage over time is displayed in Fig 6. The time-course plot suggested that the only trial-type in which bilinguals were substantially better than English speakers at identifying the target picture was different-tone trials in the Mandarin context. To investigate this pattern statistically, as with accuracy, we conducted ANOVAs on gaze.

As with gaze, we first conducted a preliminary by-subjects ANOVA with factors Trial Type, Language Context, Group, and the additional between-subjects factor of Language Order (Mandarin training first vs. English training first), in order to ascertain whether differences between bilinguals and English speakers might have been suppressed by learning English-like words before Mandarin-like words. There was a significant Language Order x Language Context interaction (F(1,75) = 10.44, *p* < .005). Listeners who learned the English-like wordset first outperformed listeners who learned Mandarin-like words first, but only for the Mandarin-like wordset. However, since there was no significant interaction between Language Order and Group (bilinguals vs. English speakers), the ANOVAs reported below collapse across Language Order for simplicity. with factors Trial Type, Language Context, and Group.

The gaze ANOVA revealed a main effect of Language Context (F1(1,77) = 22.94, *p* < .001; F2(1,15) = 11.70, *p* < .005), reflecting higher overall target-advantage scores in the Mandarin context (*M*, 0.35, *SD*, 0.18) than in the English context (*M*, 0.26, *SD*, 0.17). A main effect of Group (F1(1,77) = 7.12, *p* < .01; F2(1,15) = 17.79, *p* = .001) reflected higher overall target-advantage scores among bilinguals (*M*, 0.34, *SD*, 0.18) than among English speakers (*M*, 0.25, *SD*, 0.19).

Of particular interest was the 3-way interaction between Group, Language Context, and Trial Type, which was significant by subjects (F1(1,77) = 3.99, *p* < .05) but not by items (F2(1,15) = 1.11, *p* = .31). However, because we had an a priori interest in the Language Context by Language Background interaction in tone trials specifically, we conducted separate ANOVAs for each trial type, with factors Language Context and Group. The ANOVA for different-vowel trials showed a significant main effect of Language Context (F1(1,77) = 19.78, *p* < .001; F2(1,15) = 14.74, *p* < .005), indicating higher target fixation in the Mandarin context than the English context, and a marginal effect of Group (F1(1,77) = 3.42, *p* = .068; F2(1,15) = 6.00, *p* < .05), indicating slightly higher overall target fixation for bilinguals than English speakers. However, the two factors did not significantly interact.

The ANOVA for tone trials showed a main effect of Group (F1(1,77) = 6.49, *p* < .05; F2(1,15) = 19.69, *p* < .001), again indicating higher target fixation for bilinguals than English speakers. Importantly, there was also an interaction of Language Context and Group (F1(1,77) = 7.92, *p* < .01; F2(1,15) = 4.34, *p* = .055). Follow-up comparisons revealed that bilinguals’ target-advantage scores in tone trials significantly exceeded English speakers’ in the Mandarin context (t1(77) = 3.44, *p* = .001; t2(15) = 3.87, *p* < .005; significant after Bonferroni correction) but not in the English context (n.s.; see Table 11 for means).

**Table 11:** Means (SDs) for target advantage across language groups and trial types in Experiment 3.

	English context		Mandarin context	
	Bilinguals	English speakers	Bilinguals	English speakers
**Baseline trials**	0.39 (0.16)	0.37 (0.13)	0.44 (0.16)	0.33 (0.16)
**Different-tone&vowel trials**	0.33 (0.16)	0.28 (0.15)	0.41 (0.17)	0.32 (0.17)
**Different-vowel trials**	0.29 (0.17)	0.22 (0.18)	0.40 (0.16)	0.33 (0.17)
**Different-tone trials**	0.27 (0.16)	0.24 (0.19)	0.38 (0.18)	0.22 (0.19)
**All trials**	0.32 (0.17)	0.28 (0.17)	0.41 (0.17)	0.30 (0.18)

#### Effects of language dominance on bilinguals’ accuracy and gaze

As in the previous experiments, to address whether performance amongst bilinguals varied with proficiency in Mandarin, we conducted ANCOVAs on accuracy and gaze with Mandarin Dominance as a predictor. Unlike in Experiment 1, we found no effects of or interactions with Mandarin Dominance (n.s.).

### Discussion

In the current experiment, we found that bilinguals processed tone more efficiently than English speakers, but *only* in the Mandarin context. Bilinguals’ tone advantage in the Mandarin context suggests that they processed tone language-specifically. In other words, when learning English-like words, they processed tone no better than the English speakers did, but when learning Mandarin-like words, they used tone better than English speakers did. Accuracy and gaze patterned similarly, but effects were somewhat weaker for accuracy, potentially because training to criterion on minimal-pair contrasts may have led to ceiling effects.

This result replicates the finding from Experiments 1 and 2 that tone-language bilinguals’ phonetic processing was superior to English speakers’ specifically for Mandarin-like words and not for English-like words. However, recall that in Experiment 1, bilinguals outperformed English speakers for both tones and vowels. In Experiment 3, we equated vowel content and tone contours (via resynthesis) across English-like and Mandarin-like wordsets, and words differed across languages only in the presence or absence of final consonants. In this case, bilinguals showed an advantage for Mandarin-like words that appears to be specific to *tone* processing. However, this conclusion must be somewhat tentative given the lack of a significant 3-way interaction of trial-type, language background, and language context in the by-item ANOVA. It is possible that the small number of items (16) reduced the power of the by-item analysis.

Unlike in Experiment 1, bilinguals’ degree of Mandarin dominance did not predict their word-learning accuracy. This is especially striking because, for the participants in Experiment 3, Mandarin dominance *did* influence both accuracy and gaze efficiency in the familiar-word post-test (replicating work reported elsewhere [47]). The lack of Mandarin-dominance effects on recognition of newly learned words suggests that explicit direction to attend to tone contrasts may have taken advantage of residual plasticity in tone encoding in less-Mandarin-dominant participants, allowing them to excel relative to English speakers. However, this overall boost for bilinguals did not occur for the English wordset. Though the same minimal tone pairs and feedback were present for the English wordset, they did not increase bilinguals’ encoding of tone in that condition relative to English speakers.

## General Discussion

We asked how listeners with or without tone-language experience—Mandarin-English bilinguals vs. English speakers—encoded and processed lexical tones and vowels in novel words taught in English vs. Mandarin language contexts. We considered two types of language context: global, extra-word language context and within-word phonetic/phonological context.

In Experiment 1, global language context did not impact tone use, but tone-word learning was affected by listeners’ experience with Mandarin, with Mandarin-English bilinguals out-performing English speakers on both tone and vowel contrasts. In addition, Mandarin dominance on the MINT vocabulary test was correlated with word-learning accuracy.

Experiment 2 investigated *within-word* phonetic/phonological context, contrasting the Mandarin-compatible words from Experiment 1 with words containing strongly English-like segments and phonotactics. Bilinguals no longer out-performed English speakers, and Mandarin dominance no longer predicted accuracy. Cross-experiment analyses confirmed that bilinguals showed accuracy and gaze advantages over English speakers only in Experiment 1 (Mandarin-compatible words), not in Experiment 2 (English-like words). Thus, across the two experiments, within-word phonological context shifted bilinguals’ processing.

Experiment 3 manipulated language context within-subjects. Bilinguals processed tone information more efficiently than English speakers only in the Mandarin context. Mandarin dominance no longer predicted word-learning accuracy, as it had in Experiment 1, suggesting that the focused practice provided by the design of Experiment 3 may have bolstered lower-Mandarin-proficiency bilinguals’ knowledge of tones. This suggests that such “rusty” bilinguals are capable of encoding Mandarin-like tone-containing words as well as more-proficient bilinguals, if their attention is drawn to tone via explicit instruction and feedback on minimal pairs, consistent with previous findings of savings in phonetic relearning [53, 54].

The present findings have implications for the question of whether pitch-processing experience generalizes across languages and across cognitive domains, or is instead highly context-specific. In the Introduction, we discussed evidence for reciprocal facilitation of lexical tone and musical pitch ([22, 25, 29]), suggesting generalization of pitch processing across domains. By contrast, our findings suggest that bilinguals’ processing of pitch content is tailored to the language context. Discrimination ability alone cannot account for our effect, as our finding is not that bilinguals process pitch more effectively *overall* than English speakers, but that they do so specifically when the context supports tone processing—when it is phonologically compatible with Mandarin.

### Within-word context: how does it work?

Across the three experiments, our findings suggest that within-word context may be more consequential than extra-word context for language-specific phonological encoding. There are multiple potential explanations for our finding that bilinguals out-performed English speakers in tone processing for Mandarin-like words but not for English-like words. One possibility raised in the Introduction was that the phonetic content of words in general might *cue* bilinguals that they were in a Mandarin or an English context, making them attend or not attend to pitch accordingly. While this account could explain why a Mandarin-like wordset led to greater use of tone than an English-like wordset, it also would have predicted an effect of the extra-word phonetic cues in the original broader-language-context manipulation, so that the language in which the experiment was conducted should have served as a cue to shift bilinguals’ tone processing across Experiments 1 and 2. We found no such effect.

A second explanation, which appears to be more compatible with our findings, is that listeners encoded Mandarin-sounding novel words in a Mandarin-like way because of their holistic similarity to Mandarin-sounding words in the listener’s lexicon. That is, Mandarin-sounding novel words were pulled into the Mandarin “cluster” in lexical memory, and English-sounding words were pulled into the English cluster, without any need for top-down context to cue attentional shifts to particular phonetic properties. Within this account, words could be analyzed according to dimensional weights specific to the cluster, so that pitch contour is highly weighted in the Mandarin-like cluster(s) but not the English-like cluster(s). Either top-down contextual cueing or clustering based on phonetic distributions would enable differences in listeners’ reference frames (either phonetic context, or lexical-phonetic distributions) for different languages. Thus—crucially—they would both allow bilinguals to process phonetic information, including tone, in accordance with the phonological system of the particular language.

English speakers’ behavior in our experiments also supports the notion that bilinguals tailor their phonetic processing to the language context. Laboratory exposure to words with tone content did not enable English speakers in Experiment 1 or in Experiment 3’s Mandarin context to use tone as well as bilinguals. This confirms that our context effect for bilinguals reflects something about their *representations* of the two languages, not merely a local perceptual-learning effect [11, 12]. English speakers’ disadvantage for tones in Mandarin words is expecially interesting in that tones were a valid cue for differentiating words—particularly in Experiment 3, when many words contrasted only in tone. In other tasks, such as learning words from different talkers [41, 43], listeners *do* seem to use additional acoustic cues to recognize words in real time, even though these cues are not used to distinguish words in their language. The present findings add to the evidence that non-tone-language speakers are less sensitive to tones [19, 20, 55, 56], or process them differently [57, 58], than tone-language speakers. The present study also contributes the novel finding that bilinguals only show a tone-processing advantage over English speakers in a Mandarin context, suggesting that bilinguals modulate tone processing to match the phonetic structure of the each of their languages.

### Limitations and alternative explanations

In Experiment 3, when vowel content was closely equated between wordsets, we found a processing advantage for bilinguals in tone trials, but not vowel trials. Nevertheless, the lack of the full interaction pattern (not significant by-items) advocates for some caution before concluding that bilinguals’ advantage is specific to tone. One might infer from our findings that bilinguals have a general advantage for Mandarin-compatible words, rather than specifically a tone advantage. We found consistently better performance by bilinguals in the more phonetically Mandarin contexts, but not the Englishlike contexts. It may be that, for bilinguals, even vowel contrasts are more easily processed in a Mandarin-like frame.

Nevertheless, it may be important that in all three experiments, tone content was better equated across the two wordsets than vowel content. Even in Experiment 3, when every effort was made to equate vowels across the language stimuli, it was not always possible to use identical vowel phonemes in the two languages (see Table 9). In addition, because the wordsets (between Experiment 1 and Experiment 2 and between the two language contexts of Experiment 3) were produced by different speakers, one with a Mandarin accent and one with an American English accent, the vowels likely contained subphonemic, accentual information that was compatible with Mandarin and English, respectively. This could have contributed to a slight vowel-processing advantage for bilinguals over English speakers for the Mandarin wordsets of Experiments 1 and 3.

Another intriguing possibility is that English speakers’ difficulty differentiating tones might actually make vowel-disambiguated trials more difficult for them, by introducing more potential auditory competitors for the target word. For example, when hearing “dei2” in the presence of the “dja2” object, English speakers might activate dei4 (even though it is visually absent) more than bilingual listeners do. This would slow down overall processing, given evidence that eye-gaze is slowed by visually absent competitors ([59, 60]).

## Conclusion

We found that bilinguals who speak both a tone and an intonation language processed tone in a language-specific manner. Changing the phonetic and phonotactic content of the word itself modulated bilinguals’ use of tone, but conducting the experiment in Mandarin vs. English did not, suggesting that within-word phonetic/phonological content shifts bilinguals’ processing more than extra-word (global) context. These results extend the literature on the role of language context in bilinguals’ sound processing to lexical-tone processing, demonstrating that bilinguals can process tone in accordance with the language context.

## Supporting Information

S1 Supplemental Materials Brief descriptions of four supplemental experiments (Experiments A, B, C, and D) referred to in the main text. (DOCX)

## References

[1] Huang L. M. (1992). Remarks on the phonological structure of Mandarin Chinese. Bulletin of National Taiwan Normal University, 37, 363–383.

[2] Howie J. M. (1976). Acoustical studies of Mandarin vowels and tones. Cambridge: Cambridge University Press.

[3] McCawley J. D. (1978). What is a tone language? In Fromkin V. A., (Ed.), *Tone*: *A Linguistic Survey*, New York: Academic Press, pp. 113–131.

[4] Sebastián-Gallés N., Echeverría S., & Bosch L. (2005). The influence of initial exposure on lexical representation: Comparing early and simultaneous bilinguals. *Journal of Memory and Language*, 52, 240–255.

[5] MacWhinney B. (2008). A unified model of language acquisition In Kroll J. F. & DeGroot A., (Eds.), *Handbook of Bilingualism*: *Psycholinguistic Approaches*, pp. 49–67.

[6] De Bot K. (1992). A bilingual production model: Levelt's 'speaking' model adapted. *Applied Linguistics*, 13, 1–24.

[7] Gonzales K., & Lotto A. J. (2013). A bafri, un pafri: Bilinguals’ pseudoword identifications support language-specific phonetic systems. *Psychological Science*, 24, 2135–2142. 10.1177/0956797613486485 24022652

[8] Elman J. L., Diehl R. L., & Buchwald S. E. (1977). Perceptual switching in bilinguals. *The Journal of the Acoustical Society of America*, 62, 971–974.

[9] Flege J. E., & Eefting W. (1987). Cross-language switching in stop consonant perception and production by Dutch speakers of English. *Speech Communication*, 6, 185–202.

[10] Hazan V. L., & Boulakia G. (1993). Perception and production of a voicing contrast by French-English bilinguals. *Language and Speech*, 36, 17–38.

[11] Bohn O.-S., & Flege J. (1988). Perceptual switching in Spanish/English bilinguals. *The Journal of the Acoustical Society of America*, 83, S27–S27.

[12] Garcia-Sierra A., Diehl R. L., & Champlin C. (2009). Testing the double phonemic boundary in bilinguals. *Speech Communication*, 51, 369–378. 10.1016/j.specom.2008.11.005 19829747 PMC2760981

[13] Kraljic T., & Samuel A. G. (2006). Generalization in perceptual learning for speech. *Psychonomic Bulletin & Review*, 13, 262–268. 16892992 10.3758/bf03193841

[14] Godden D. R., & Baddeley A. D. (1975). Context-dependent memory in two natural environments: On land and underwater. *British Journal of Psychology*, 66, 325–331.

[15] Marian V., & Neisser U. (2000). Language-dependent recall of autobiographical memories. *Journal of Experimental Psychology*: *General*, 129, 361. 11006905 10.1037//0096-3445.129.3.361

[16] Singh L., & Quam C. (2016). Can bilingual children turn one language off? Evidence from perceptual switching. *Journal of Experimental Child Psychology*, 147, 111–125. 10.1016/j.jecp.2016.03.006 27077335

[17] Mattock K., & Burnham D. (2006). Chinese and English infants' tone perception: Evidence for perceptual reorganization. *Infancy*, 10, 241–265.

[18] Yeung H. H., Chen K. H., & Werker J. F. (2013). When does native language input affect phonetic perception? The precocious case of lexical tone. *Journal of Memory and Language*, 68, 123–139.

[19] Krishnan A., Xu Y., Gandour J., & Cariani P. (2005). Encoding of pitch in the human brainstem is sensitive to language experience. *Cognitive Brain Research*, 25, 161–168. 10.1016/j.cogbrainres.2005.05.004 15935624

[20] Chandrasekaran B., Krishnan A., & Gandour J. T. (2007). Experience-dependent neural plasticity is sensitive to shape of pitch contours. *Cognitive Neuroscience and Neuropsychology*, 18, 1963–1967. 10.1097/WNR.0b013e3282f213c5 PMC4374445 18007195

[21] Hallé P. A., Chang Y.-C., & Best C. T. (2004). Identification and discrimination of Mandarin Chinese tones by Mandarin Chinese vs. French listeners. *Journal of Phonetics*, 32, 395–421.

[22] Pfordresher P. Q., & Brown S. (2009). Enhanced production and perception of musical pitch in tone language speakers. *Attention*, *Perception*, *& Psychophysics*, 71, 1385–1398. 10.3758/APP.71.6.1385 19633353

[23] Bidelman G. M., Hutka S., & Moreno S. (2013). Tone language speakers and musicians share enhanced perceptual and cognitive abilities for musical pitch: evidence for bidirectionality between the domains of language and music. *PLoS One*, 8, e60676 10.1371/journal.pone.0060676 23565267 PMC3614545

[24] Hove M. J., Sutherland M. E., & Krumhansl C. L. (2010). Ethnicity effects in relative pitch. *Psychonomic Bulletin & Review*, 17, 310–316. 20551351 10.3758/PBR.17.3.310

[25] Chandrasekaran B., Krishnan A., & Gandour J. T. (2009). Relative influence of musical and linguistic experience on early cortical processing of pitch contours. *Brain and Language*, 108, 1–9. 10.1016/j.bandl.2008.02.001 18343493 PMC2670545

[26] Lee, J., Perrachione, T. K., Dees, T. M., & Wong, P. C. (2007). Differential effects of stimulus variability and learners’ pre-existing pitch perception ability in lexical tone learning by native English speakers. In 16th International Congress of Phonetic Sciences.

[27] Wong P. C., & Perrachione T. K. (2007). Learning pitch patterns in lexical identification by native English-speaking adults. *Applied Psycholinguistics*, 28, 565–585.

[28] Wong P. C., Skoe E., Russo N. M., Dees T., & Kraus N. (2007). Musical experience shapes human brainstem encoding of linguistic pitch patterns. *Nature Neuroscience*, 10, 420–422. 10.1038/nn1872 17351633 PMC4508274

[29] Alexander, J. A., Wong, P. C., & Bradlow, A. R. (2005). Lexical tone perception in musicians and non-musicians. In Proceedings of the 9th European Conference on Speech Communication and Technology. Lisbon.

[30] Burnham, D., Francis, E., Webster, D., Luksaneeyanawin, S., Lacerda, F., & Attapaiboon, C. (1996). Facilitation or attenuation in the development of speech mode processing? Tone perception over linguistic contexts. In Proceedings of the Sixth Australian International Conference on Speech Science and Technology.

[31] Quam C., & Swingley D. (2010). Phonological knowledge guides 2-year-olds’ and adults’ interpretation of salient pitch contours in word learning. *Journal of Memory and Language*, 62, 135–150. 10.1016/j.jml.2009.09.003 20161601 PMC2811275

[32] Quam C., & Swingley D. (2012). Development in children’s interpretation of pitch cues to emotions. *Child Development*, 83, 236–250. 10.1111/j.1467-8624.2011.01700.x 22181680 PMC3397680

[33] Díaz B., Mitterer H., Broersma M., & Sebastián-Gallés N. (2012). Individual differences in late bilinguals' L2 phonological processes: From acoustic-phonetic analysis to lexical access. *Learning and Individual Differences*, 22, 680–689.

[34] Bidelman G. M., Gandour J. T., & Krishnan A. (2011). Musicians and tone-language speakers share enhanced brainstem encoding but not perceptual benefits for musical pitch. *Brain and Cognition*, 77, 1–10. 10.1016/j.bandc.2011.07.006 21835531 PMC3159732

[35] Chandrasekaran B., Sampath P. D., & Wong P. C. (2010). Individual variability in cue-weighting and lexical tone learning. *The Journal of the Acoustical Society of America*, 128, 456–465. 10.1121/1.3445785 20649239 PMC2921440

[36] Gollan T. H., Weissberger G. H., Runnqvist E., Montoya R. I., & Cera C. M. (2012). Self-ratings of spoken language dominance: A multi-lingual naming test (MINT) and preliminary norms for young and aging Spanish-English bilinguals. *Bilingualism*, 15, 594 10.1017/S1366728911000332 25364296 PMC4212892

[37] Dunn A. L., & Fox Tree J. E. (2009). A quick, gradient bilingual dominance scale. *Bilingualism*: *Language and Cognition*, 12, 273–289.

[38] Creel S. C., Aslin R. N., & Tanenhaus M. K. (2006). Acquiring an artificial lexicon: Segment type and order information in early lexical entries. *Journal of Memory and Language*, 54, 1–19.

[39] Shen X. S., & Lin M. (1991). A perceptual study of Mandarin tones 2 and 3. *Language and Speech*, 34, 145–156.

[40] Boersma, P., & Weenink, D. (2009). Praat: Doing phonetics by computer (Version 5.1. 07) [Computer program]. Retrieved May 1, 2009, http://www.praat.org/.

[41] Creel S. C., Aslin R. N., & Tanenhaus M. K. (2008). Heeding the voice of experience: The role of talker variation in lexical access. *Cognition*, 106, 633–664. 10.1016/j.cognition.2007.03.013 17507006

[42] Creel S. C., Tanenhaus M. K., & Aslin R. N. (2006). Consequences of lexical stress on learning an artificial lexicon. *Journal of Experimental Psychology*: *Learning*, *Memory*, *and Cognition*, 32, 15 10.1037/0278-7393.32.1.15 16478337

[43] Creel S. C., & Tumlin M. A. (2011). On-line acoustic and semantic interpretation of talker information. *Journal of Memory and Language*, 65, 264–285.

[44] Brainard D. H. (1997). The psychophysics toolbox. *Spatial Vision*, 10, 433–436. 9176952

[45] Pelli D. G. (1997). The VideoToolbox software for visual psychophysics: Transforming numbers into movies. *Spatial Vision*, 10, 437–442. 9176953

[46] Cornelissen F. W., Peters E. M., & Palmer J. (2002). The Eyelink Toolbox: Eye tracking with MATLAB and the Psychophysics Toolbox. *Behavior Research Methods*, *Instruments*, *& Computers*, 34, 613–617. 10.3758/bf03195489 12564564

[47] Quam C., & Creel S. (in press). Tone attrition in Mandarin speakers of varying English proficiency. *Journal of Speech*, *Language*, *and Hearing Research*. 10.1044/2016_JSLHR-S-15-0248 PMC5533551 28124064

[48] Wendt D., Brand T., & Kollmeier B. (2014). An eye-tracking paradigm for analyzing the processing time of sentences with different linguistic complexities. *PloS One*, 9, e100186 10.1371/journal.pone.0100186 24950184 PMC4065036

[49] Barr D. J. (2008). Analyzing ‘visual world’ eyetracking data using multilevel logistic regression. *Journal of Memory and Language*, 59, 457–474.

[50] Hallett P. E. (1986). Eye movements In Boff K. R., Kaufman L., & Thomas J. P., (Eds.), *Handbook of Perception and Human Performance*, New York: Wiley,

[51] Kaushanskaya M., & Marian V. (2009). The bilingual advantage in novel word learning. *Psychonomic Bulletin & Review*, 16, 705–710. 19648456 10.3758/PBR.16.4.705

[52] Muench K. L., & Creel S. C. (2013). Gradient phonological inconsistency affects vocabulary learning. *Journal of Experimental Psychology*: *Learning*, *Memory*, *and Cognition*, 39, 1585 10.1037/a0032862 23647379

[53] Bowers J. S., Mattys S. L., & Gage S. H. (2009). Preserved implicit knowledge of a forgotten childhood language. *Psychological Science*, 20, 1064–1069. 10.1111/j.1467-9280.2009.02407.x 19645694

[54] Au T. K.-f., Knightly L. M., Jun S.-A., & Oh J. S. (2002). Overhearing a language during childhood. *Psychological Science*, 13, 238–243. 12009044 10.1111/1467-9280.00444

[55] Liang J., & van Heuven V. J. (2007). Chinese tone and intonation perceived by L1 and L2 listeners. *Tones and Tunes*, 2, 27–61.

[56] Gottfried T. L., & Suiter T. L. (1997). Effect of linguistic experience on the identification of Mandarin Chinese vowels and tones. *Journal of Phonetics*, 25, 207–231.

[57] Wang Y., Jongman A., & Sereno J. A. (2001). Dichotic perception of Mandarin tones by Chinese and American listeners. *Brain and Language*, 78, 332–348. 10.1006/brln.2001.2474 11703061

[58] Wang Y., Behne D. M., Jongman A., & Sereno J. A. (2004). The role of linguistic experience in the hemispheric processing of lexical tone. *Applied Psycholinguistics*, 25, 449–466.

[59] Magnuson J. S., Tanenhaus M. K., Aslin R. N., & Dahan D. (2003). The time course of spoken word learning and recognition: Studies with artificial lexicons. *Journal of Experimental Psychology*: *General*, 132, 202. 12825637 10.1037/0096-3445.132.2.202

[60] Kapnoula E. C., Packard S., Gupta P., & McMurray B. (2015). Immediate lexical integration of novel word forms. *Cognition*, 134, 85–99. 10.1016/j.cognition.2014.09.007 25460382 PMC4255136

