# Supplementary material for: Mandarin-English Bilinguals Process Lexical Tones in Newly Learned Words in Accordance with the Language Context
Source: PLoS One. 2017 Jan 11;12(1):e0169001. doi: 10.1371/journal.pone.0169001 (PMC5226804; doi:10.1371/journal.pone.0169001)
Supplement: S1 Supplemental Materials — (DOCX) [file pone.0169001.s001.docx]

**Supplemental Materials**

***Experiment A***

To confirm that our speaker had native-like proficiency in Mandarin, we intermixed her 16 English-carrier sentences (“Choose the [biu3fu]”) with corresponding sentences recorded by four English speakers with no tone-language training and one very Mandarin-dominant speaker from mainland China (with a BDS score of 13). Fourteen bilingual participants rated each sentence on how much the talker “sounds like they are from China or Taiwan” (on a scale from 0 to 1) and “sounds like they are from the US” (also on a scale from 0 to 1). Our speaker’s compatibility with Chinese accent was rated as 0.78, and her compatibility with American accent only 0.25, making her much more likely to be from China or Taiwan than the US (paired t(13) = 5.37; p < .001). The difference score (Chinese rating minus English rating) for our speaker (0.53) was quite similar to that of the Mandarin-dominant speaker (0.52; unpaired t(13) = -0.07, p = 0.94), but was significantly different from the ratings for each of the four English speakers (-.08, -.39, -.44, and -.21; all unpaired t(13) > 5; all p < .001).

***Experiment B***

To gauge whether our Experiment 1 novel words were more Mandarin-like than English-like on some dimension, despite our efforts to balance the phonotactics between Mandarin and English, we conducted a rating task with 14 additional bilingual listeners (the same as in *Experiment A*). An American-English speaker produced the original sixteen words, along with three more foil versions of each word that contained increasingly more English-like segments, providing a range of Mandarin- vs. English-sounding words (note that the most English-like words were used as stimuli in Experiment 2; and are listed in **Table 6** of the main document). Ratings of Mandarin-likeness and English-likeness indicated that the Experiment 1 novel words were, as designed, fairly equally compatible with both Mandarin and English. There was a trend for higher ratings of Mandarin-likeness than English-likeness (mean difference, .10; t(13) = 2.05, *p* = .061) when words contained Mandarin tones, but not when words were spoken with neutral English (rise-fall) intonation instead of Mandarin tones (mean difference, 0.01), suggesting that the difference was driven by tonal information.

***Experiment C***

A gating experiment with twelve bilingual adults assessed whether tone differences were detectable later in our stimuli than vowel differences. Listeners heard successively longer portions of the words from Experiment 1 (increasing by 40 ms). All gates began at the onset of the sentence and then ended at some point in the target word. The first gate ended before the onset of the first vowel (e.g., the /i/ in “fi4pu”), and gates were added until the end of the first syllable was reached; the final gate presented the entire word. Participants were asked to click on the spelling that best matched the truncated word. For seven participants familiar with Pinyin, we used Pinyin spellings; for five participants not familiar with Pinyin, we used English-like spellings. Accuracy averaged over the first 9 gates (before words were fully disambiguated) was included in by-subject and by-item ANOVAs with Trial Type (tone- or vowel-disambiguated) as a within-subject and within-item categorical predictor. These revealed a significant main effect of Trial Type (F1(1,11) = 17.93, *p* = .001; F2(1,15) = 31.22, *p* < .001); accuracy was significantly higher in vowel-disambiguated (*M =* 86%, *SD* = 5%) than tone-disambiguated trials (*M* = 77%, *SD* = 10%). This result suggests that the Experiment 1 disadvantage for tone processing relative to vowel processing was primarily due to tones being disambiguated later in the signal than vowels.

***Experiment D***

To verify that the tones in the Experiment 2 English-like words were equivalent to the tones in the Experiment 1 words, we conducted a brief rating experiment with 12 additional bilingual listeners on extracted pitch contours from the two sets of recordings. Listeners identified the isolated pitch contours from four options (Tones 1–4). Tone identification was above chance (that is, above 25%; t(11) = 6.08, *p* < .001) and did not differ between Experiment 1 (61%) and Experiment 2 words (58%; paired t(11) = 0.94, *p* = 0.37). Nearly half (46%) of tone confusions were between tones 2 and 3, which, because of their high confusability, were intentionally never paired in test trials. If Tone 2 was counted as an acceptable response in Tone 3 trials and vice-versa, accuracy increased to 78% in both conditions. Ratings of goodness, computed over correct trials only, were also comparable for the two word-sets (Experiment 1, 66.5%; Experiment 2, 67.2%; paired t(11) = -0.24, *p* = 0.82), suggesting that the two speakers produced the pitch contours of Mandarin tones with comparable precision.
